# Knockout of Hepatocyte Growth Factor by CRISPR/Cas9 System Induces Apoptosis in Hepatocellular Carcinoma Cells

**DOI:** 10.3390/jpm11100983

**Published:** 2021-09-29

**Authors:** Han Ki Lee, Heui Min Lim, See-Hyoung Park, Myeong Jin Nam

**Affiliations:** 1 Department of Biological Science, Gachon University, Seongnam 13120, Korea; akdmadl94@naver.com (H.K.L.); chrislim36@gmail.com (H.M.L.); 2 Department of Bio and Chemical Engineering, Hongik University, Sejong 30016, Korea

**Keywords:** CRISPR/Cas9 system, hepatocyte growth factor, hepatocellular carcinoma, apoptosis, c-Met signaling pathway, mitogen activated protein kinases

## Abstract

**Background**: CRISPR/Cas9 system is a prokaryotic adaptive immune response system that uses noncoding RNAs to guide the Cas9 nuclease to induce site-specific DNA cleavage. Hepatocyte growth factor (HGF) is a well-known growth factor that plays a crucial role in cell growth and organ development. According to recent studies, it has been reported that HGF promoted growth of hepatocellular carcinoma (HCC) cells. Here, we investigated the apoptotic effects in HCC cells. **Methods:** Crispr-HGF plasmid was constructed using GeneArt CRISPR Nuclease Vector. pMex-HGF plasmid that targets HGF overexpressing gene were designed with pMex-neo plasmid. We performed real time-polymerase chain reaction to measure the expression of HGF mRNA. We performed cell counting assay and colony formation assay to evaluate cell proliferation. We also carried out migration assay and invasion assay to reveal the inhibitory effects of Crispr-HGF in HCC cells. Furthermore, we performed cell cycle analysis to detect transfection of Crispr-HGF induced cell cycle arrest. Collectively, we performed annexin V/PI staining assay and Western blot assay. **Results:** In Crispr-HGF-transfected group, the mRNA expression levels of HGF were markedly downregulated compared to pMex-HGF-transfected group. Moreover, Crispr-HGF inhibited cell viability in HCC cells. We detected that wound area and invaded cells were suppressed in Crispr-HGF-transfected cells. The results showed that transfection of Crispr-HGF induced cell cycle arrest and apoptosis in HCC cells. Expression of the phosphorylation of mitogen activated protein kinases and c-Met protein was regulated in Crispr-HGF-transfected group. Interestingly, we found that the expression of HGF protein in conditioned media significantly decreased in Crispr-HGF-transfected group. **Conclusions:** Taken together, we found that inhibition of HGF through transfection of Crispr-HGF suppressed cell proliferation and induced apoptotic effects in HCC Huh7 and Hep3B cells.

## 1. Introduction

Hepatocellular carcinoma (HCC) is known to be the most common disease in some areas of Asia and Africa [1]. The prevalence of HCC is increasing rapidly in the western countries such as Europe and America [2]. HCC is emerging as the leading cause of death in males, especially in developing countries [3]. Hence, HCC is being concentrated as a medical issue in the world. Many treatments such as chemotherapy and radiofrequency ablation for HCC have been developed. However, these therapies have some drawbacks, such as detrimental effects of radiation therapy on normal liver tissue [4]. Thus, it is necessary to develop alternative therapeutic method to treat HCC [5]. It is reported that the clustered regularly interspaced short palindromic repeats (CRISPR)-associated (Cas) protein 9 (CRISPR/Cas9) system is currently in the spotlight for gene therapy purposes [6]. This technique can be a novel therapy for curing hepatocellular carcinoma. Therefore, HGF protein or HGF gene therapy may improve conventional treatment.

Hepatocyte growth factor (HGF) is a multifunctional growth factor which is secreted by mesenchymal stem cells that play crucial roles in accelerating the invasion and metastasis of several cancer cells [7]. HGF and its receptor c-Met plays an important role in cancer growth and metastasis [8]. HGF/c-Met signaling is drawing attention as an important target molecule for anticancer therapy because it is related to cancer cell generation, growth, invasion, and metastasis, and various small molecule and antibody therapeutics are being developed [9]. In terms of tumor microenvironment, HGF creates an environment favorable for the growth of cancer cells through interaction with cancer cells [10]. HGF regulates the sugar metabolism pattern of cancer cells to induce the growth of cancer cells [11]. It also induces the transformation of the tumor microenvironment into an immune-suppressing environment through metabolites [12]. As such, HGF/c-Met signaling plays an important role in tumor immune evasion and resistance to treatment in the tumor microenvironment.

The CRISPR/Cas9 system was discovered as an acquired immune system, and it was observed in bacteria and archaea [13]. In CRISPR/Cas9 system, invading external DNA is processed by Cas9 nuclease into small DNA fragments, which are then incorporated into CRISPR locus of host genomes as the spacers [14]. CRISPR/Cas9 system has become a potent method for making alterations to the genome of many organisms [15]. According to recent studies, it is reported that the CRISPR/Cas9 system promises to promote cancer research by offering an efficient technology to analysis mechanisms of tumorigenesis, identifying targets for drug development, and possibly arming cells for cell-based therapies [15]. Thus, the mechanism of CRISPR/Cas9 system has been studied in detail and it currently being used as a gene editing tool. This system allows the target gene expression to be regulated by 20 nucleotide sequences [13].

In this study, we evaluated if the transfection of Crispr-HGF gene using the CRISPR/Cas9 system has potential roles in treating HCC in vitro. The objective of our study was to demonstrate the apoptotic effects of transfection of Crispr-HGF in HCC Huh7 and Hep3B cells and describe the functions of Crispr-HGF in downregulating HGF protein in conditioned media.

## 2. Materials and Methods

### 2.1. Chemical Reagents and Antibodies

Human HGF were purchased from R&D systems. Akt, pAkt, ERK, pERK, E-cadherin, N-cadherin, vimentin, PARP, caspase-3, Bim, and Bax primary antibodies were purchased from Cell Signaling Technology (Danvers, MA, USA). β-actin, p38, pp38, JNK, pJNK, Bcl-xL, and HGF primary antibodies were obtained from Santa Cruz Biotechnology (Santa Cruz, CA, USA). Met and pMet primary antibodies were purchased from Young-In frontier (Seoul, Korea). Goat anti-mouse and goat anti-rabbit horseradish secondary antibodies were purchased from Cell Signaling Technology (Danvers, MA, USA). Primary antibodies were used in 1:1000 dilution and secondary antibodies were used in 1:10,000 dilution. The primers were obtained from Bioneer (Seongnam, Korea).

### 2.2. Cell Culture

Hepatocellular carcinoma Huh7 and Hep3B cells were obtained from Korea Cell Line Bank (Seoul, Korea). Huh7 and Hep3B cells were maintained under standard conditions (5% CO_2_, 37 °C, and 95% humidity). Huh7 and Hep3B cells were cultured in Dulbecco’s Modified Eagle Medium (DMEM) (GIBCO, Grand Island, NY, USA) media containing 10% heat-inactivated fetal bovine serum (Young in Frontier) and 1% penicillin/streptomycin (GIBCO). Both cell lines were used at passages 4–20 for all experiments.

### 2.3. Plasmid Construction

**Figure 1:**
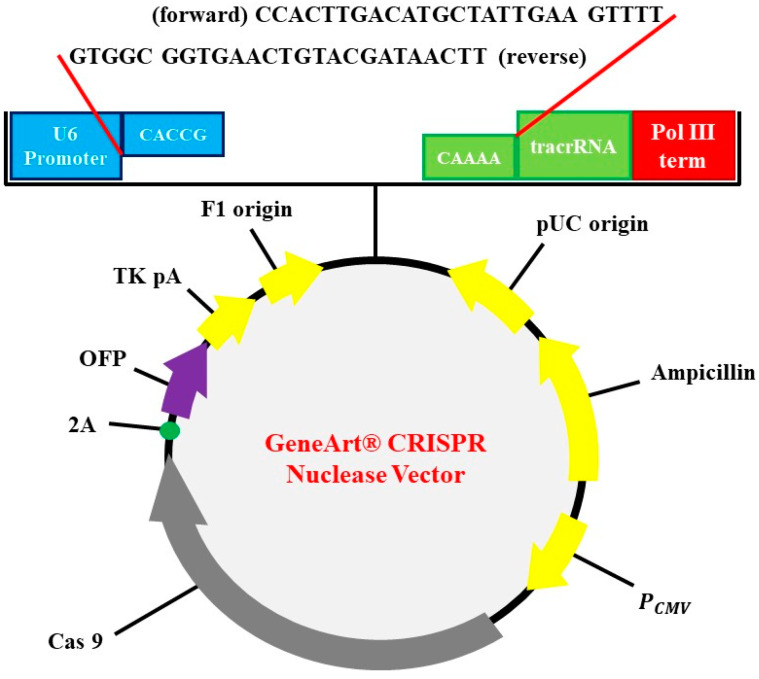
Identification of plasmid. CRISPR-HGF plasmid vector map and 20 base nucleotides of sgRNA insertion. It is referred to by previous research [16]. The diagram represents vector map of CRISPR/Cas9 vector, GeneArt CRISPR Nuclease vector (9219 bp). The guide RNA (gRNA) ligation was enabled by using BbsI as a restriction enzyme. U6 promoter, orange fluorescent protein (OFP), CMV enhancer, gRNA scaffold, Cas9, and ampicillin resistant gene (AmpR) are included in the sequence elements.

The subclonal reconstruction of pMex-neo with human HGF genome using SalI restriction enzyme was used to construct pMex-HGF plasmid. GeneArt CRISPR Nuclease Vector with OFP Reporter Kit (Thermo Fisher Scientific, Waltham, MA, USA) was used to construct the HGF-CRISPR-plasmid. GeneArt CRISPR gRNA Design Tool was used for synthesizing oligonucleic acids for gRNA. The oligonucleic acid was annealed and inserted into CRISPR vector. Escherichia coli DH5α was used as transformation strain followed by antibiotics selection for preparation of vectors (Figure 1).

The following CRISPR RNA (crRNA) sequences were used to guide Cas9 to target DNA:

HGF (forward): 5′-CCACTTGACATGCTATTGAA-3′

HGF (reverse): 5′-TTCAATAGCATGTCAAGTGG-3′

### 2.4. Transfection

Huh7 and Hep3B cells (2 × 10^5^/well) were seeded in a 6-well plate and incubated for 24 h. pMex-neo, pMex-HGF, Crispr-control, and Crispr-HGF DNA were diluted (2 μg/mL) in opti-MEM media (without serum) with lipofectamine 2000 (Invitrogen, Carlsbad, California) (6 μg/mL). After gentle pipetting, pMex-neo, pMex-HGF, Crispr-control, and Crispr-HGF solutions were incubated in a water bath (37 °C) for 30 min. Both cells were treated with lipofectamine-DNA complex solution and incubated for 6–18 h. Cells were washed twice with PBS before media are replaced with DMEM growth medium containing 10% FBS and 1% antibiotics, and an additional 24, 48, and 72 h incubation was carried out for efficient expression of transfected DNA.

### 2.5. Real-Time Quantitative Reverse Transcription Polymerase Chain Reaction (qRT-PCR)

TRIzol reagent (Invitrogen) was used to extract total RNA from the transfected cells and 2 μg of total RNA was used to synthesize the first-strand cDNA using oligo (dT) Maxime RT premix kit (iNtRON Biotechnology, Seongnam, Korea). Real-time reverse transcription-polymerase chain reaction (qRT-PCR) was carried out according to the manufacturer’s instructions (iNtRON Biotechnology) using 20 μL reactions containing 2 μL of cDNA template, 10 μL QuantiSpeed SYBR (Philekorea Technology, Seoul, Korea) and 2 μM of each following primer:

HGF

Forward: 5′-ATGATGATGCTCATGGACCCT-3′

Reverse: 5′-CTGGCAAGCTTCATTAAAACC-3′

GAPDH

Forward: 5′-TGCACCACCAACTGCTTAGC-3′

Reverse: 5′-TGGCATGGACTGTGGTCATGA-3′

The procedure started with initial denaturation step (95 °C for 10 min) followed by a two-step PCR (20 sec at 95 °C; 1 min at 60 °C, 40 cycles). The relative amount of HGF mRNA was normalized with corresponding Ct values of endogenous control, Glyceraldehyde 3-phosphate dehydrogenase (GAPDH), mRNA using the 2^−^^∆∆^^Ct^ method.

### 2.6. Conditioned Media Preparation

Huh7 and Hep3B cells were seeded at 100 mm cell culture dish and incubated under standard conditions (5% CO_2_, 37 °C, and 95% humidity) and transfected Crispr-Control and Crispr-HGF. after PBS washing. Subsequently, the cultured serum free media were centrifuged to remove cell debris and supernatant were transferred to Amicon Ultra-15 centrifugal filter (Millipore, Billerica, MA, USA). Bradford assay was performed to quantify protein concentration. Conditioned media were stored at −80 °C.

### 2.7. Cell Counting Assay

Huh7 and Hep3B cells (2 × 10^5^) were seeded at 6-well cell culture dish to attach and transfected Crispr-Control and Crispr-HGF. After 24, 48, and 72 h, the cells were harvested using 0.25% trypsin-EDTA, respectively. At each day, the cell numbers were counted by using hemocytometer.

### 2.8. Colony Formation Assay

Huh7 and Hep3B cells were seeded at 6-well cell culture dish at a density of 1 × 10^3^ cells and incubated under standard conditions (5% CO_2_, 37 °C, and 95% humidity) to attach and transfected Crispr-Control and Crispr-HGF. Then, the media was replaced with fresh media containing 10% FBS and cells were incubated for 15 days, replacing the fresh media containing 10% FBS once every 3 days under standard conditions. The cells were washed with PBS and fixed using 4% formaldehyde for 30 min at 4 °C. Subsequently, the cells were washed PBS again and stained for 1 h with 1% crystal violet (Sigma, St. Louis, MO, USA) solution, and then the colonies were counted.

### 2.9. Wound Healing Assay

This assay was performed to identify whether Crispr-HGF suppressed cancer cell migration in cells. Huh7 and Hep3B cells were seeded at 6-well plates at a density of 2 × 10^5^ cells and incubated under standard conditions (5% CO_2_, 37 °C, and 95% humidity) until the cells were reached 90% confluence. Then, the center of the well was scratched with the P200 pipette tip and the cells were rinsed twice with PBS to remove the cell debris. Subsequently, the cells were transfected Crispr-Control and Crispr-HGF. The wound area closure was observed using a fluorescence microscope (CKX53; Olympus, Tokyo, Japan).

### 2.10. Invasion Assay

6.5 mm transwell with 8.0 μm pore size polyester membrane insertion (Corning Inc., Corning, NY, USA) was used for invasion assay. The lower sides of the sterile 8 μm polyethylene filters were pre-coated with gelatin (1 mg/mL: Sigma). The filters were hydrated with 100 μL serum-free DMEM media and 24-well plate were filled with 750 μL DMEM media with serum (10% FBS). The gelatin-coated transwell chambers were filled with additional 200 μL serum-free DMEM media containing cells (2.5 × 10^5^ cells/mL), transfected with Crispr-control or Cripsr-HGF vectors and additionally cultured for 24, 48, and 72 h. The transwell chambers were placed on 24-well plate and incubated at standard condition (37 °C in a humidified incubator containing 5% CO_2_ in air). After 24 h incubation, the media were removed and transwell chambers were rinsed twice with PBS. Cells were fixed with 3.7% formaldehyde for 20 min at room temperature and then permeabilized with 100% methanol for 20 min. After fixation and permeabilization procedure, cells were rinsed with PBS 2 times and dyed with Giemsa stain (Sigma) for 30 min. The cells were rinsed twice with PBS and the cells on the upper surface of the filter were completely removed gently with a cotton swab. The images of the migrated cells were taken using Nikon Eclipse TE 2000-U (Tokyo, Japan) at 200× magnification.

### 2.11. Cell Cycle Analysis

Huh7 and Hep3B cells were seeded in 6-well cell culture dish at a density of 2 × 10^5^ cells and incubated at 37 °C overnight to attach. The cells were transfected with Crispr-HGF for 24, and 48 h. Following that, cells were harvested using by 0.25% trypsin-EDTA and fixed using cold 70% EtOH for 2 h at 4 °C. Subsequently, cells were centrifuged at 1350 rpm for 3 min and incubated with PI (Sigma) (50 μg/mL PI and 200 μg/mL RNase A) for 30 min at room temperature in dark room. The cell cycle distribution data were measured by flow cytometry (Beckman Coulter, CA, USA).

### 2.12. Annexin V/PI Staining Assay

Huh7 and Hep3B cells were seeded in 6-well plates (2 × 10^5^/well) and cultured until the cell confluency reaches 70%. The cells were transfected with Crispr-control or Crispr-HGF vectors for 18 h and media were changed to fresh DMEM media with 10% FBS. After 24, 48, and 72 h additional cultures, apoptotic rates were examined by using Annexin V-FITC Apoptosis Staining/Detection Kit (Abcam, Cambridge, MA, USA). Huh7 and Hep3B cells were also transfected with pMex-neo, pMex-HGF, Crispr-control and Crispr-HGF and treated with H_2_O_2_ (400 μM) for 24 h after 72 h of additional incubation and the apoptotic rates were examined. The cells were washed with PBS twice and harvested using trypsin-EDTA (Sigma). After centrifugation, the supernatant was removed, and the cells were resuspended in 500 µL of 1× binding buffer (5 µL of Annexin V-FITC and 5 µL propidium iodide added) and incubated for 5 min. Apoptotic cells were determined by flow cytometry (Beckman Coulter Brea, Brea, CA, USA).

### 2.13. Western Blot Assay

Huh7 and Hep3B cells were transfected with pMex-neo, pMex-HGF, Crispr-control and Crispr-HGF for 18 h and cultured for 72 h. Additional treatment with or without H_2_O_2_ (400 μM) for 24 h was applied to cultured transfected cells. The cells were harvested and lysed in Radioimmunoprecipitation assay (RIPA) buffer, protease inhibitor (Sigma) and phenylmethylsulfonylfluoride (Sigma). Concentration of proteins were measured by Bradford assay. Protein samples were resolved by SDS-PAGE (Sodium dodecyl sulfate polyacrylamide gel electrophoresis) at 120 V for 2 h and transferred to methanol-activated polyvinylidene fluoride membrane at 50 V for 2 h. The gels were stained using coomassie blue (Bio-sesang, Seong-Nam, Korea) for overnight. The membranes were then blocked using 3% bovine serum albumin (BSA) for 30 min at 4 °C and incubated with specific primary antibodies at 4 °C overnight. Then the membranes were washed with TBS-T three times every 10 min and incubated with secondary antibodies (Santa Cruz, Dallas, TX, USA). The membranes were washed with TBS-T once again and chemiluminescence was measured using detection reagents (GE healthcare, Little Chalfont, UK) and Chemi-doc detection system (Bio-rad, Hercules, CA, USA).

### 2.14. Statistical Analysis

The results are expressed as arithmetic mean + standard deviation. To compare the data between the groups, two-sided unpaired Student’s t-test was used. Experiments were repeated three times, and the representative data were shown. A one-way ANOVA followed by Bonferroni post hoc test was used for statistical analysis and a *p* value of <0.05 was considered statistically significant.

## 3. Results

### 3.1. Transfection of Crispr-HGF Downregulated the Expression Levels of HGF mRNA in Huh7 and Hep3B Cells

**Figure 2:**
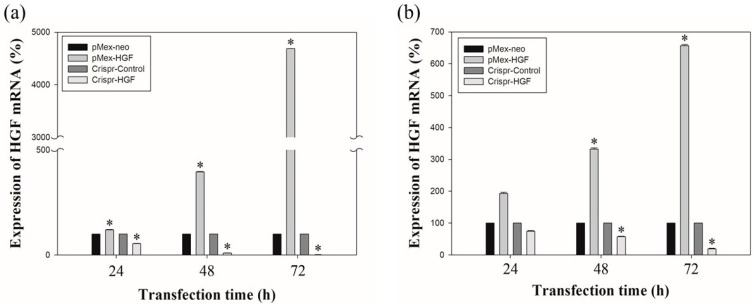
The mRNA expression levels of HGF as measured by qRT-PCR. Huh7 and Hep3B cells were transfected with the pMex-neo or pMex-HGF, and Crispr-Control or Crispr-HGF plasmid. Total mRNA was isolated from the cells 24, 48, and 72 h after transfection. (**a**) HGF mRNA increased in pMex-HGF-transfected cells, however, decreased in Crispr-HGF-transfected Huh7 cells. (**b**) In same condition, we also determine HGF mRNA increased in pMex-HGF-transfected cells, however, decreased in Crispr-HGF-transfected Hep3B cells. Error bar represents the mean ± SEM. * *p* < 0.05. These assays were performed at least three times in triplicate.

The results of real time-PCR showed that transfection of Crispr-HGF reduced HGF mRNA expression levels. Transfection with pMex-HGF plasmid increased the HGF mRNA levels to 117.6%, 395.4%, and 4685.1%, compared to Huh7 cells transfected with pMex-neo plasmid at 24, 48, and 72 h after incubation, respectively. In Hep3B cells, HGF mRNA levels were increased to 193.2%, 331.7%, and 655.8%, respectively, in pMex-HGF-transfected cells, compared to pMex-neo-transfected cells. Nevertheless, the HGF mRNA levels of transfected with Crispr-HGF plasmid decreased to 54.3%, 9.5%, and 0.5%, respectively, compared to the Huh7 cells transfected with Crispr-Control plasmid. Additionally, in Hep3B cells, the mRNA levels were decreased to 74.6%, 58.9%, and 21% in Crispr-HGF-transfected cells compared to Crispr-control-transfected cells, respectively (Figure 2). These results suggested that the transfection of Crispr-HGF downregulated the expression levels of HGF mRNA in Huh7 and Hep3B cells.

### 3.2. HGF Stimulated c-Met Signaling Pathways and Transfection of Crispr-HGF Regulated the Expression of HGF and MAPKs in Huh7 and Hep3B Cells

**Figure 3:**
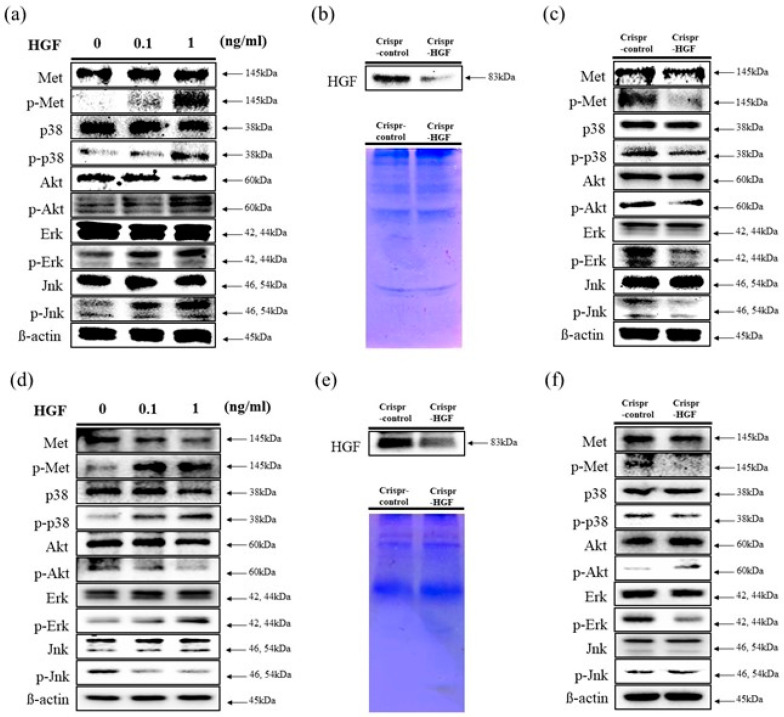
Stimulation of c-Met signaling pathways by HGF. Western blot assay was performed to verify human HGF stimulates c-Met signaling pathways. (**a**,**d**) HGF was treated with Huh7 and Hep3B cells for 1 h. The expression levels of c-Met, Akt, and several MAP kinases such as Erk, Jnk, and p38 were maintained regardless of HGF concentrations. However, the expression levels of phosphorylated status of those proteins were changed as HGF concentrations increases. β-actin was used as a loading control. (**b**,**e**) Downregulation of HGF secreted in conditioned media. We performed Western blot assay to evaluate the expression levels of HGF in conditioned media. Both cells were transfected with Crispr-Control or Crispr-HGF plasmid for 72 h. Subsequently, the Western blot assay results indicated that secreted HGF in conditioned media were decreased in Crispr-HGF group compared to Crispr-Control group. Coomassie Blue staining of SDS-PAGE were used for the loading control. (**c**,**f**) The expression levels of p-Met, p-Akt, p-Erk, p-Jnk, and p-p38 were also changed in Crispr-HGF group compared to Crispr-Control group. β-actin was used as a loading control.

We performed a Western blot assay to determine human HGF stimulates c-Met signaling pathways in Huh7 and Hep3B cells. We investigated the expression levels of c-Met proteins and MAP (Mitogen-activated protein) kinases. The results showed that the expression levels of c-Met, p38, Akt, Erk, and Jnk proteins were maintained regardless of HGF dose in both cell lines. However, the expression levels of phosphorylated c-Met, p38, Akt, Erk, and Jnk increased as HGF concentration increased in Huh7 cells. Meanwhile, in Hep3B cells, the expression levels of phosphorylated c-Met, p38, and Erk increased and that of Akt and Jnk decreased (Figure 3a,d). We also detected HGF secreted expression levels to cell culture conditioned media. In both cell lines, at 72 h of transfection time, HGF expression levels were decreased in Crispr-HGF-transfected Huh7 and Hep3B cells (Figure 3b,e). Furthermore, we measured the expression levels of c-Met proteins and several MAPKs after 72 h of transfection time through Western blot. The results showed that the expression levels of phosphorylated c-Met, p38, Akt, Erk, and Jnk decreased in Huh7 cells. However, in Hep3B cells, the expression levels of phosphorylated c-Met, p38, and Erk downregulated and that of Akt and Jnk upregulated (Figure 3c,f). Therefore, these results elucidated that HGF stimulated c-Met signaling pathways and transfection of Crispr-HGF modulated the HGF and MAPKs expression in Huh7 and Hep3B cells.

### 3.3. Transfection of Crispr-HGF Inhibited the Cell Proliferation in Huh7 and Hep3B Cells

**Figure 4:**
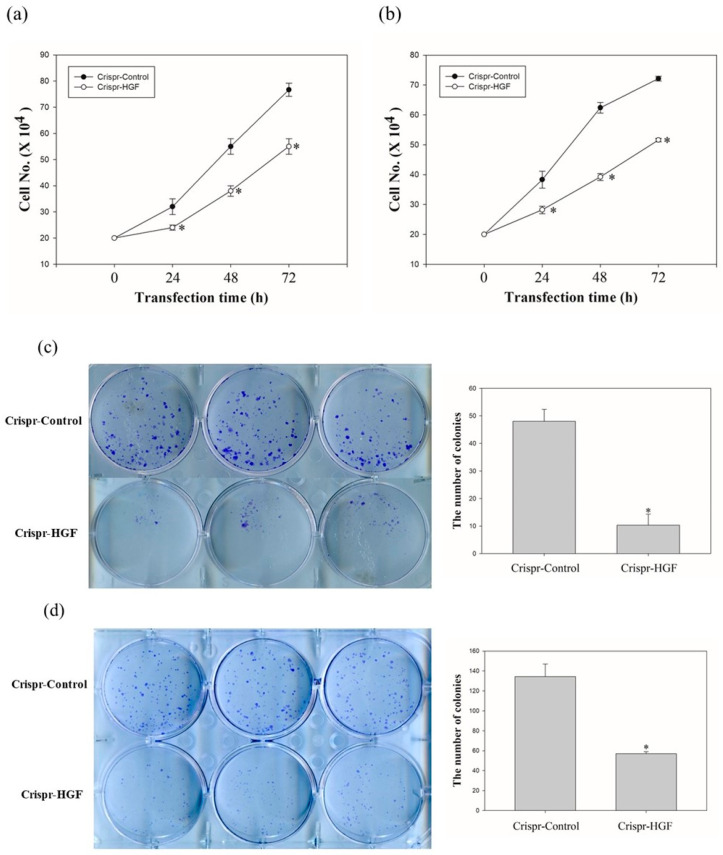
The number of (**a**) Huh7 cells and (**b**) Hep3B cells transfected with Crispr-Control or Crispr-HGF for 24, 48, and 72 h. Cell survival was determined by cell counting assay. Error bar represents the mean ± SEM. * *p* < 0.05. Colony formation assay of (**c**) Huh7 and (**d**) Hep3B cells downregulation of HGF. The numbers of colony were represented in bar graphs. Error bar represents the mean ±SEM. * *p* < 0.05. These assays were performed at least three times in triplicate.

We investigated the cell proliferation after Crispr-HGF-transfected cells via cell counting assay and colony formation assay. The proliferative ability of Huh7 and Hep3B cells following HGF knockout decreased markedly when compared to that of the respective Crispr-control-transfected cells, as detected using a cell counting assay (Figure 4a,b). Moreover, the colony formation assay revealed that the number of colonies that formed in Huh7 and Hep3B cells was downregulated when compared with cells transfected with Crispr-control vectors (Figure 4c,d). Thus, these results mean that Crispr-HGF induced the inhibition of cell proliferative ability in Huh7 and Hep3B cells.

### 3.4. Transfection of Crispr-HGF Suppressed Migration and Invasion Ability in Huh7 and Hep3B Cells

**Figure 5:**
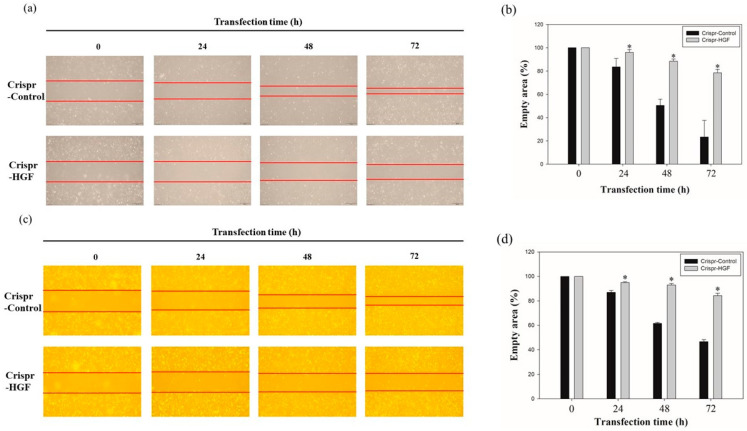
Migration ability of (**a**) Huh7 and (**c**) Hep3B cells transfected with Crispr-Control and Crispr-HGF. Wound healing assay was performed to determine cell migration ability in Huh7 and Hep3B cells transfected with Crispr-Control and Crispr-HGF in a time-dependent manner. Artificial wound area in Crispr-Control-transfected cells were covered rapidly than Crispr-HGF-transfected cells. (**b**,**d**) The bar graph showed empty area for each group according to the transfection time. Error bar represents the mean ±SEM. * *p* < 0.05. These assays were performed at least three times in triplicate.

**Figure 6:**
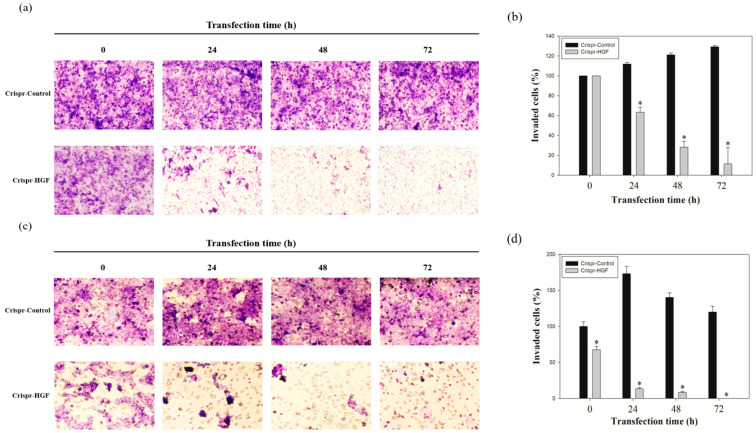
Invasion ability of (**a**) Huh7 and (**c**) Hep3B cells transfected with Crispr-Control and Crispr-HGF. Invaded cells were increased in Crispr-Control-transfected group, however, that of Crispr-HGF-transfected group were decreased. (**b**,**d**) The bar graph indicates that invaded cells were decreased in Crispr-HGF-transfected Huh7 cells. Error bar represents the mean ±SEM. * *p* < 0.05. These assays were performed at least three times in triplicate.

**Figure 7:**
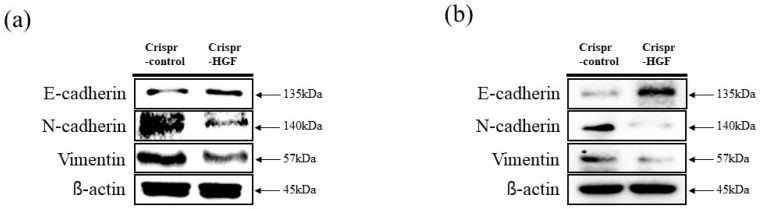
The expression levels of proteins related to cell migration through Western blot assay. The cells were transfected with Crispr-Control and Crispr-HGF for 72 h. The expression levels of E-cadherin were increased in Crispr-HGF-transfected (**a**) Huh7 and (**b**) Hep3B cells compared to control group. However, the expression levels of N-cadherin and Vimentin were decreased in Crispr-HGF-transfected cells compared to control group in both cells.

We performed wound healing assay to investigate transfection of Crispr-HGF suppressed migration ability in Huh7 and Hep3B cells. The results of wound healing assay showed that transfection of Crispr-HGF inhibited migration in a time-dependent manner. The wound area was healed more than 60% after 72 h in the Crispr-Control group in both cell lines. However, the migration ability in the Crispr-HGF transfected group was significantly decreased (Figure 5a,c). The bar graph indicates that the empty area was significantly increased in Crispr-HGF-transfected group compared to that in the Crispr-Control group (Figure 5b,d). In addition, we performed invasion assay to detect the invasive ability of Huh7 and Hep3B cells after Crispr-HGF transfection. We found that the percentage of invaded cells were decreased 63.8%, 28.1%, and 11.5% after 24, 48, and 72 h, respectively, of Crispr-HGF transfection in Huh7 cells. In Hep3B cells, the percentage of invaded cells were decreased 13.3%, 8.3%, and almost 0% after 24, 48, and 72 h, respectively, of Crispr-HGF transfection (Figure 6a,c). The bar graph indicated that the number of invaded cells was significantly decreased in Crispr-HGF-transfected Huh7 and Hep3B cells (Figure 6b,d). Furthermore, we also detected the expression levels of proteins relate to migration through Western blot assay. The expression levels of E-cadherin were increased in both Crispr-HGF-transfected cells for 72 h. The expression levels of N-cadherin, and Vimentin were significantly decreased in both Crispr-HGF-transfected cells for 72 h (Figure 7a,b). Therefore, these results demonstrated that transfection of Crispr-HGF inhibited the migration and invasion ability in Huh7 and Hep3B cells.

### 3.5. Transfection of Crispr-HGF Induced Cell Cycle Arrest in Huh7 and Hep3B Cells

**Figure 8:**
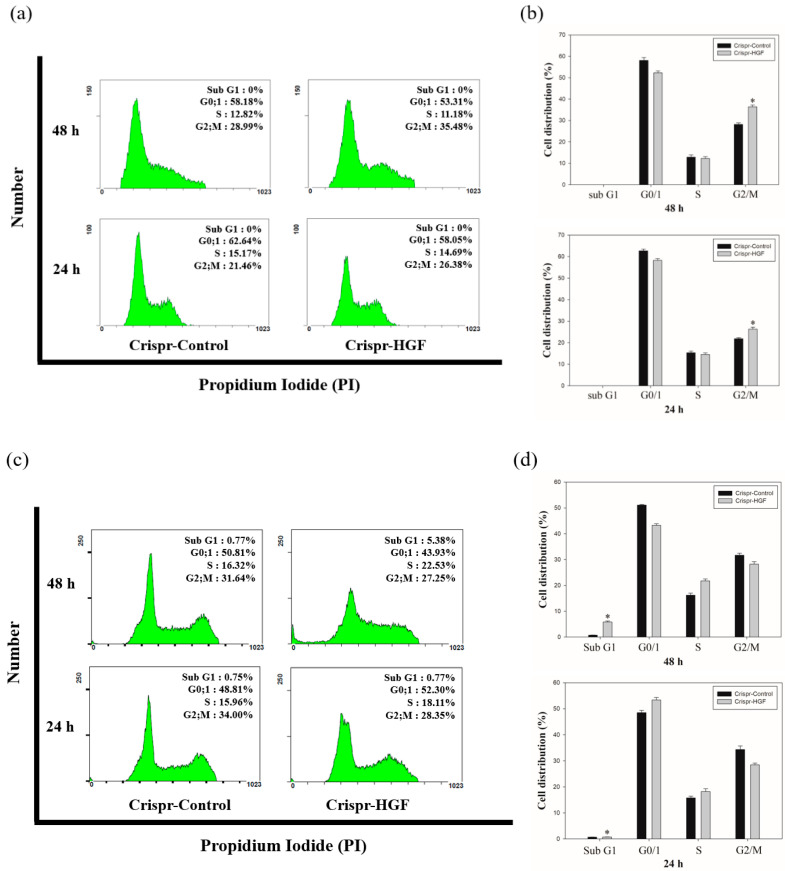
Cell cycle arrest in Crispr-HGF-transfected Huh7 and Hep3B cells. The cells were transfected with Crispr-Control and Crispr-HGF for 24 and 48 h and stained with propodium iodide (PI). After staining, the cells were detected using flow cytometry. The distribution and cell percentages of each cell cycle phase were evaluated. (**a**) G2/M cell cycle arrest in a time-dependent manner against Huh7 cells. (**c**) G0/1 cell cycle arrest and subG1 cell cycle arrest in a time-dependent manner against Hep3B cells. (**b**,**d**) The results were analyzed statistically using the Student’s *t*-test and the graph indicated the cell cycle distribution for each group. Error bar represents the mean ±SEM. * *p* < 0.05. These assays were performed at least three times in triplicate.

We performed cell cycle analysis to investigate whether Crispr-HGF transfection induced cell cycle arrest in Huh7 and Hep3B cells. The results of cell cycle analysis showed that Crispr-HGF induced G2/M cell cycle arrest in Huh7 cells. At 24 and 48 h of transfection time, we measured that G2/M phase increased by 4.92% and 6.49%, respectively (Figure 8a). Meanwhile, in Hep3B cells, Crispr-HGF induced G1 cell cycle arrest and sub-G1 cell cycle arrest. At 24 h of transfection time, we measured that G1 phase increased by 3.49% and subsequently 48 h of transfection time, sub G1 phase increased by 4.61% (Figure 8b). Furthermore, the bar graph indicated that each cell cycle distribution was represented in a time-dependent manner (Figure 8c,d). Thus, these results suggested that transfection of Crispr-HGF induced G2/M cell cycle arrest in Huh7 cells and G1 and sub G1 cell cycle arrest in Hep3B cells.

### 3.6. Transfection of Crispr-HGF Induced Apoptosis in Huh7 and Hep3B Cells

**Figure 9:**
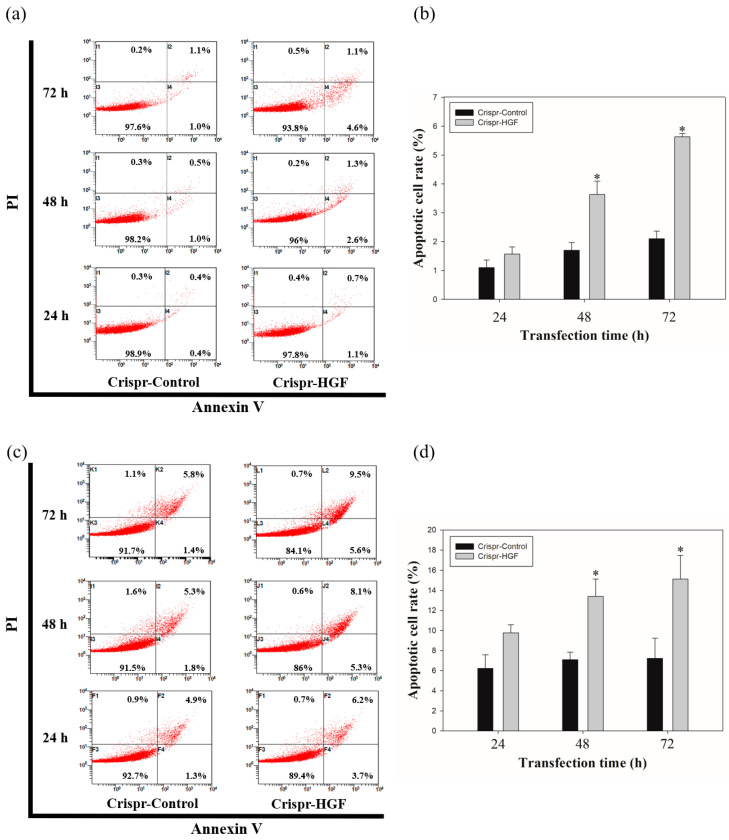
Apoptosis in Crispr-HGF-transfected (**a**) Huh7 and (**c**) Hep3B cells. Annexin V/PI staining assay determine if Crispr-HGF induces apoptosis in both cells in a time-dependent manner. Apoptotic cells rate was increased in Crispr-HGF group compared to Crispr-Control group in a time-dependent manner. (**b**,**d**) The graph showed apoptotic cell rate for each group according to transfection time. Error bar represents the mean ±SEM. * *p* < 0.05. These assays were performed at least three times in triplicate.

We measured annexin V/PI staining assay to investigate whether apoptosis progressed with Crispr-HGF transfection. We detected the total apoptotic cell rate increased according to transfection time. The apoptotic cell rate in Crispr-Control-transfected Huh7 cells were almost similar regardless of transfection time. However, we verified the apoptotic cell rate in Crispr-HGF-transfected Huh7 cells were increased by 1.8%, 3.9%, and 5.7% as the transfection time increased to 24, 48, and 72 h, respectively. We also revealed that the apoptotic cell rate in Crispr-HGF-transfected Hep3B cells were increased by 9.9%, 13.4%, and 15.1% as the transfection time increased to 24, 48, and 72 h, respectively. (Figure 9a,c). The bar graph indicated that the apoptosis rate increased visually (Figure 9b,d). Therefore, HGF gene knockout by CRSISPR/Cas9 system induces apoptosis against Huh7 and Hep3B cells in a time-dependent manner.

### 3.7. H_2_O_2_ Induced Synergistic Apoptotic Effects with Transfection of Crispr-HGF against Huh7 and Hep3B Cells

**Figure 10:**
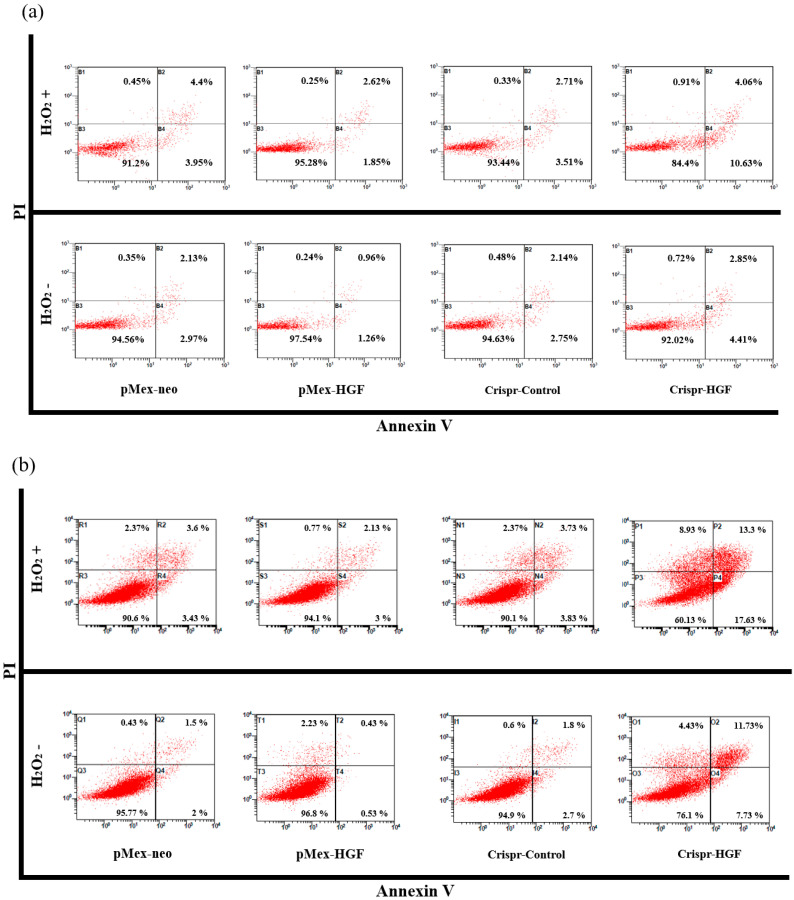

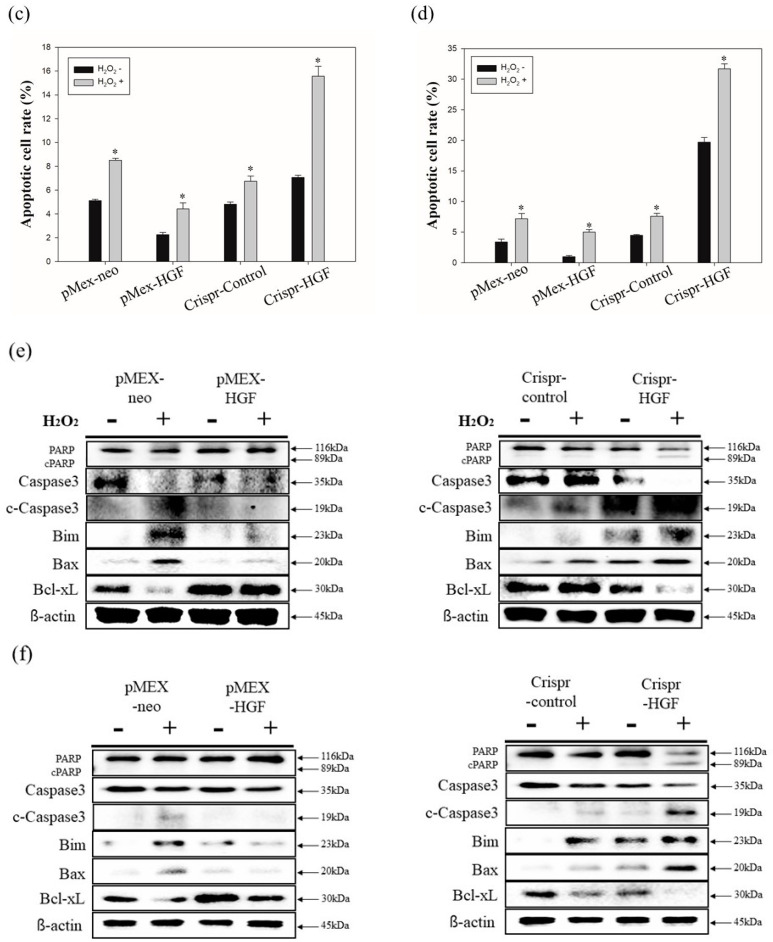
Synergistic apoptotic effects of H_2_O_2_ and Crispr-HGF. Annexin V/PI staining assay determine if the induction of apoptosis by transfection of Crispr-HGF and treatment of H_2_O_2_ in (**a**) Huh7 and (**b**) Hep3B cells. Apoptotic cells rate was increased by 9.8% in Crispr-HGF group and H_2_O_2_ treated group compared to Crispr-Control group in Huh7 cells (**c**). In addition, apoptotic cells rate was increased by 26.43% in Crispr-HGF group and H_2_O_2_ treated group compared to Crispr-Control group in Hep3B cells (**d**). Error bar represents the mean ± SEM. * *p* < 0.05. These assays were performed at least three times in triplicate. Western blot assay was performed to detect proteins relate to apoptosis in (**e**) Huh7 and (**f**) Hep3B cells. The expression levels of PARP, and caspase3 and those of cleaved forms were detected. Furthermore, the expression levels of Bim, Bax, and Bcl-xL were detected. β-actin was used as a loading control.

We performed annexin V/PI staining assay to demonstrate transfection of Crispr-HGF and H_2_O_2_ induced synergistic apoptotic effects in Huh7 and Hep3B cells. The results showed that apoptosis rate is significantly increased in both transfected with Crispr-HGF and treated with H_2_O_2_ group compared to in Crispr-Control-transfected group. Apoptotic rates were increased to 7.43% in Huh7 cells and 11.47% in Hep3B cells, respectively (Figure 10a,b). The bar graph indicated that the apoptosis rate increased visually (Figure 10c,d). Moreover, we performed Western blot assay to investigate apoptotic mechanisms of Crispr-HGF and H_2_O_2_. The results showed that the expression levels of caspase3, and PARP proteins were decreased in both transfected with Crispr-HGF and treated with H_2_O_2_ group. However, the expression levels of cleaved forms of those proteins were increased in both transfected with Crispr-HGF and treated with H_2_O_2_ group. Furthermore, the expression levels of Bim and Bax were increased in Crispr-HGF and H_2_O_2_ group, however, Bcl-xL expression levels were decreased in Crispr-HGF and H_2_O_2_ group (Figure 10e,f). Through these results, we evaluated that Crispr-HGF and H_2_O_2_ induced synergistic apoptotic effects in HCC Huh7 and Hep3B cells.

## 4. Discussion

Hepatocellular carcinoma (HCC) is one of the main causes of death in the United States as well as globally [17]. Recently, conventional therapies such as chemotherapy, surgery, and radiation offer impoverished treatment options [18]. However, there are still many side effects of the current cancer therapies against advanced metastasized cancer [19]. This is why many scientists and doctors have been anxious to discover alternative methods to treat HCC [18]. Therefore, as of now, a different treatment for hepatocellular carcinoma should be sought. Among them, gene therapy such as CRISPR/Cas9 system should be studied in more depth.

Hepatocyte growth factor (HGF) is known as scatter factor that is involved in various cell regeneration, including in the central nervous system [20]. It also referred to as scatter factor, and it was first identified and purified from serum and plasma as a potent mitogen for hepatocytes in 1984 [21]. Subsequently, HGF was confirmed in several other organs, such as kidney, heart, and liver [22]. HGF is a well-known powerful pleiotropic cytokine that is entailed in mitogenesis, morphogenesis, and angiogenesis and anti-apoptosis in lots of cells and tissue reproductions [23]. HGF was identified as a novel growth factor with unique structural characteristics [24]. HGF is secreted as a single-chain, inactive polypeptide by mesenchymal stem cells and is cleaved to its active extracellular forms by numerous proteases [25]. HGF is synthesized and secreted as a single-chain inactive precursor by mesodermal origin cells, and extracellularly processed to the two-chain functional heterodimer by proteolytic cleavage at a specific site [26]. The binding of HGF to the c-Met, the HGF receptor, induces activation of tyrosine kinase and autophosphorylation of tyrosine residues [27]. c-Met activation propagates an intricate system of signaling pathways that regulates a range of cellular process as diverse as cell proliferation, differentiation, transformation, and apoptotic cell death [28]. Based on our studies, qRT-PCR results suggested that the expression levels of HGF are dramatically increased in HGF-overexpression transfection group, whereas that of Crispr-HGF-transfected group is significantly decreased in both Huh7 and Hep3B cell lines (Figure 2a,b).

Mitogen activated protein kinase (MAPK) is composed of three well-known subfamilies namely, extracellular signal-regulated kinases (Erk), c-Jun amino-terminal kinases (Jnk), and p38 [29]. These MAPKs are entailed in directing cellular responses to a diverse array of stimuli, such as mitogens, osmotic stress, heat shock, and proinflammatory [30]. Erks control cell separation, Jnk is involved in transcription, and p38 regulate differentiation, apoptosis, and autophagy [31]. It is reported in several studies that MAPK plays an important role in the growth and survival of liver cancer cells [32]. MAPKs and Akt are direct downstream of c-Met signaling pathway and involved in cell sustenance [33]. Based on our studies, we considered that HGF mediates regulation of MAPKs as well as c-Met signaling pathways. Our results suggested that human HGF affects upregulation of phosphorylated Akt, Erk, Jnk, p38, and c-Met expression levels in Huh7 cells (Figure 3a). Additionally, in Crispr-HGF-transfected group, the expression levels of these phosphorylated MAPKs decreased as well as phosphorylated c-Met in Huh7 cells (Figure 3c). However, interestingly, in Hep3B cells, human HGF led to accumulation of phosphorylated c-Met, p38, and Erk expression levels and dissipation of phosphorylated Akt and Jnk expression levels (Figure 3d). In addition, the expression levels of phosphorylated c-Met, p38, and Erk decreased and the expression levels of phosphorylated Akt and Jnk increased in Crispr-HGF-transfected Hep3B cells (Figure 3f). Furthermore, the expression levels of HGF in Crispr-HGF-transfected group were significantly decreased in both cell lines (Figure 3b,e). Therefore, HGF gene therapies should be more emphasized in curing HCC.

It is reported that overexpression of HGF can cause renal cell carcinoma, gastric cancer, and HCC and may also stimulate the growth of hidden cancer cells [34]. As shown in Figure 4, cell counting results suggested that cell numbers increased rapidly in Crispr-Control-transfected group compared to Crispr-HGF-transfected group in Huh7 and Hep3B cells. Moreover, colony formation results demonstrated that Crispr-HGF inhibited colony forming ability significantly in Huh7 and Hep3B cells. These results suggested that knockout of HGF through transfection of Crispr-HGF gene induces cell growth inhibition in Huh7 and Hep3B cells.

Transfection of Crispr-HGF induces inhibition of cell migration and invasion in Huh7 and Hep3B cells. It is reported that HGF stimulation increased cellular migration, downregulation of the expression levels of E-cadherin, and upregulation of the expression levels of N-cadherin and vimentin in HCC cell lines [35]. Our results suggested that cell migration ability decreased significantly in Crispr-HGF-transfected group compared to Crispr-Control-transfected group in a time dependent manner (Figure 5). Likewise, invaded cells dramatically decreased in Crispr-HGF-transfected group compared to Crispr-Control group in a time-dependent manner (Figure 6). Furthermore, transfection of Crispr-HGF increased the expression levels of E-cadherin and decreased the expression levels of N-cadherin and vimentin (Figure 7). Therefore, these results are a measure that transfection of Crispr-HGF could induce apoptotic effects in HCC cells by reducing the migration and invasion ability.

Induction of HGF/cMet inhibition resulted in phase arrest of cell cycle and apoptosis in numerous cancer cell species, including primary effusion lymphoma [36], multiple myeloma [37], and gastric cancer [38]. Here, downregulation of HGF expression via Crispr-HGF transfection was used to achieve the analysis of cell cycle arrest on HCC. Our results showed that HGF knockout induced G2/M cell cycle arrest in Huh7 cells and G0/G1 cell cycle arrest in Hep3B cells in a time-dependent manner (Figure 8). These results provide a conclusion that is consistent with the previous result of cell counting.

It is reported that apoptosis by transfected Crispr-HGF was induced in mesenchymal stem cells [13]. Our results indicated that transfection of Crispr-HGF induces apoptosis in a time-dependent manner in Huh7 and Hep3B cells. Moreover, apoptotic cell rates increase according to transfection time 24, 48, and 72h, respectively, in both cell lines (Figure 9). Taken together, these results supported the fact that transfection of Crispr-HGF induces apoptotic effects in HCC Huh7 and Hep3B cells.

Furthermore, it is reported that H_2_O_2_ induced ROS level elevation and apoptosis in prostate cancer cells via ERK activation [39]. Previous studies represented to present that overexpression of HGF/cMet inhibited apoptosis in cancer cells via modulating mitochondrial proteins [40]. ROS-mediated apoptosis is critically associated with the intracellular ROS accumulation and regulation of the proapoptotic BH3-only proteins (Bim and Bax) and anti-apoptotic proteins (Bcl-xL) expression, which triggers mitochondrial membrane permeabilization [41,42]. Thus, we verified that transfection of Crispr-HGF and H_2_O_2_ induced apoptosis in HCC Huh7 and Hep3B cells. Apoptotic cells rate was markedly increased in both Crispr-HGF-transfected cells and the H_2_O_2_ treated group compared to Crispr-Control-transfected cells (Figure 10a,d). Moreover, we also performed Western blot assays to detect apoptotic mechanisms of Crispr-HGF and H_2_O_2_. The expression levels of Bcl-xL were decreased in treated with H_2_O_2_ and transfected with Crispr-HGF. However, the expression levels of Bax and Bim were increased in treated with H_2_O_2_ and transfected with Crispr-HGF. The expression levels of cleaved caspase3 and cleaved PARP were significantly increased in treated with H_2_O_2_ and transfected with Crispr-HGF (Figure 10e,f). In brief, these results suggest that H_2_O_2_ induced synergistic apoptotic effects with Crispr-HGF in HCC cells.

In this study, we examined the apoptotic activity of Crispr-HGF transfection in HCC, Huh7 and Hep3B cells and investigated its apoptotic mechanisms. We have revealed that the apoptotic activity of Crispr-HGF in HCC cells was mediated by downregulation of HGF expression. Fundamentally, transfection of Crispr-HGF inhibited cell growth and colony formation ability in HCC cells. Interestingly, the expression levels of secreted HGF in conditioned media were also decreased in Crispr-HGF-transfected group. Therefore, our findings suggest that regulating the expression of HGF gene through CRISPR/Cas9 system might play important roles in apoptotic effects of HCC cells. However, the experiments were done only in cellular level and further studies must be done to specifically target the cancer cells. This study is believed to provide general understanding of apoptotic mechanisms of HGF knockout in HCC and give an idea as a candidate in developing anti-cancer therapies.

## Data Availability

The datasets used in the current study are available from the corresponding author upon reasonable request.

## Abbreviations

HCC	Hepatocellular carcinoma
CRISPR/Cas9	Clustered regularly interspaced short palindromic repeats (CRISPR)-associated (Cas) protein 9 (CRISPR/Cas9) system
HGF	Hepatocyte growth factor
RIPA	Radioimmunoprecipitation assay
PARP	Poly (ADP-ribose) polymerase
Akt	Protein kinase B (PKB)
DMSO	Dimethyl sulfoxide
FBS	Fetal bovine serum
PBS	Phosphate-buffered saline
PI	Propidium iodide

## References

[1] Llovet J.M., Kelley R.K., Villanueva A., Singal A.G., Pikarsky E., Roayaie S., Lencioni R., Koike K., Zucman-Rossi J., Finn R.S. (2021). Hepatocellular carcinoma. Nat. Rev. Dis. Primers.

[2] Puoti C. (2018). New insights on hepatocellular carcinoma: Epidemiology and clinical aspects. Hepatoma Res..

[3] Lodato F., Mazzella G., Festi D., Azzaroli F., Colecchia A., Roda E. (2006). Hepatocellular carcinoma prevention: A worldwide emergence between the opulence of developed countries and the economic constraints of developing nations. World J. Gastroenterol..

[4] Moini M., Schilsky M.L., Tichy E.M. (2015). Review on immunosuppression in liver transplantation. World J. Hepatol..

[5] Kang M.A., Jeon Y.K., Nam M.J. (2020). Auricularia auricula increases an apoptosis in human hepatocellular carcinoma cells via a regulation of the peroxiredoxin1. J. Food Biochem..

[6] Akram F., Ikram Ul H., Ahmed Z., Khan H., Ali M.S. (2020). CRISPR-Cas9, A Promising Therapeutic Tool for Cancer Therapy: A Review. Protein Pept. Lett..

[7] Kuang W., Deng Q., Deng C., Li W., Shu S., Zhou M. (2017). Hepatocyte growth factor induces breast cancer cell invasion via the PI3K/Akt and p38 MAPK signaling pathways to up-regulate the expression of COX2. Am. J. Transl. Res..

[8] Lesko E., Majka M. (2008). The biological role of HGF-MET axis in tumor growth and development of metastasis. Front. Biosci..

[9] Xiang C., Chen J., Fu P. (2017). HGF/Met Signaling in Cancer Invasion: The Impact on Cytoskeleton Remodeling. Cancers.

[10] Matsumoto K., Umitsu M., De Silva D.M., Roy A., Bottaro D.P. (2017). Hepatocyte growth factor/MET in cancer progression and biomarker discovery. Cancer Sci..

[11] Boschert V., Klenk N., Abt A., Janaki Raman S., Fischer M., Brands R.C., Seher A., Linz C., Müller-Richter U.D.A., Bischler T. (2020). The Influence of Met Receptor Level on HGF-Induced Glycolytic Reprogramming in Head and Neck Squamous Cell Carcinoma. Int. J. Mol. Sci..

[12] Breunig C., Erdem N., Bott A., Greiwe J.F., Reinz E., Bernhardt S., Giacomelli C., Wachter A., Kanthelhardt E.J., Beißbarth T. (2018). TGFβ1 regulates HGF-induced cell migration and hepatocyte growth factor receptor MET expression via C-ets-1 and miR-128-3p in basal-like breast cancer. Mol. Oncol..

[13] Choi Y.J., Lee C.M., Lee J.H., Park S.H., Nam M.J. (2019). Protective effects of hepatocyte growth factor gene overexpression against hydrogen peroxide-induced apoptosis in mesenchymal stem cells. Environ. Toxicol..

[14] Loureiro A., da Silva G.J. (2019). CRISPR-Cas: Converting A Bacterial Defence Mechanism into A State-of-the-Art Genetic Manipulation Tool. Antibiotics.

[15] Zhan T., Rindtorff N., Betge J., Ebert M.P., Boutros M. (2019). CRISPR/Cas9 for cancer research and therapy. Semin. Cancer Biol..

[16] Chesnut J., Namritha R., Kumar S., Garza J. (2015). Efficient and Multiplex Genome Editing Using GeneArt® CRISPR Nuclease mRNA.

[17] Lee C.M., Lee J., Jang S.-N., Shon J.C., Wu Z., Park K., Liu K.-H., Park S.-H. (2020). 6,8-Diprenylorobol Induces Apoptosis in Human Hepatocellular Carcinoma Cells via Activation of FOXO3 and Inhibition of CYP2J2. Oxidative Med. Cell. Longev..

[18] Lee C.M., Choi Y.J., Park S.-H., Nam M.J. (2018). Indole-3-carbinol induces apoptosis in human hepatocellular carcinoma Huh-7 cells. Food Chem. Toxicol..

[19] Park S.H., Phuc N.M., Lee J., Wu Z., Kim J., Kim H., Kim N.D., Lee T., Song K.S., Liu K.H. (2017). Identification of acetylshikonin as the novel CYP2J2 inhibitor with anti-cancer activity in HepG2 cells. Phytomedicine.

[20] Li B., Leung J.C.K., Chan L.Y.Y., Yiu W.H., Li Y., Lok S.W.Y., Liu W.H., Chan K.W., Tse H.F., Lai K.N. (2019). Amelioration of Endoplasmic Reticulum Stress by Mesenchymal Stem Cells via Hepatocyte Growth Factor/c-Met Signaling in Obesity-Associated Kidney Injury. STEM CELLS Transl. Med..

[21] Zeng W., Ju R., Mao M. (2015). Therapeutic potential of hepatocyte growth factor against cerebral ischemia (Review). Exp. Med..

[22] Nakamura T., Mizuno S. (2010). The discovery of hepatocyte growth factor (HGF) and its significance for cell biology, life sciences and clinical medicine. Proc. Jpn. Acad. Ser. B Phys. Biol. Sci..

[23] Longati P., Albero D., Comoglio P.M. (1996). Hepatocyte growth factor is a pleiotropic factor protecting epithelial cells from apoptosis. Cell Death Differ..

[24] Sato H., Aoki S., Kato T., Matsumoto K., Choi S. (2018). HGF (Hepatocyte Growth Factor). Encyclopedia of Signaling Molecules.

[25] Fukushima T., Uchiyama S., Tanaka H., Kataoka H. (2018). Hepatocyte Growth Factor Activator: A Proteinase Linking Tissue Injury with Repair. Int. J. Mol. Sci..

[26] Galimi F., Cottone E., Vigna E., Arena N., Boccaccio C., Giordano S., Naldini L., Comoglio P.M. (2001). Hepatocyte Growth Factor Is a Regulator of Monocyte-Macrophage Function. J. Immunol..

[27] Zhang Y., Xia M., Jin K., Wang S., Wei H., Fan C., Wu Y., Li X., Li X., Li G. (2018). Function of the c-Met receptor tyrosine kinase in carcinogenesis and associated therapeutic opportunities. Mol. Cancer.

[28] Organ S.L., Tsao M.-S. (2011). An overview of the c-MET signaling pathway. Adv. Med. Oncol..

[29] Guo Y.J., Pan W.W., Liu S.B., Shen Z.F., Xu Y., Hu L.L. (2020). ERK/MAPK signalling pathway and tumorigenesis (Review). Exp. Med..

[30] Zhang W., Liu H.T. (2002). MAPK signal pathways in the regulation of cell proliferation in mammalian cells. Cell Res..

[31] He X., Wang C., Wang H., Li L., Wang C. (2020). The Function of MAPK Cascades in Response to Various Stresses in Horticultural Plants. Front. Plant. Sci..

[32] Huynh H., Nguyen T.T.T., Chow K.-H.K.-P., Tan P.H., Soo K.C., Tran E. (2003). Over-expression of the mitogen-activated protein kinase (MAPK) kinase (MEK)-MAPK in hepatocellular carcinoma: Its role in tumor progression and apoptosis. BMC Gastroenterol..

[33] Jung K.H., Park B.H., Hong S.S. (2012). Progress in cancer therapy targeting c-Met signaling pathway. Arch. Pharm. Res..

[34] Herrero-Fresneda I., Torras J., Franquesa M., Vidal A., Cruzado J.M., Lloberas N., Fillat C., Grinyó J.M. (2006). HGF gene therapy attenuates renal allograft scarring by preventing the profibrotic inflammatory-induced mechanisms. Kidney Int..

[35] Nagai T., Arao T., Furuta K., Sakai K., Kudo K., Kaneda H., Tamura D., Aomatsu K., Kimura H., Fujita Y. (2011). Sorafenib inhibits the hepatocyte growth factor-mediated epithelial mesenchymal transition in hepatocellular carcinoma. Mol. Cancer.

[36] Dai L., Trillo-Tinoco J., Cao Y., Bonstaff K., Doyle L., Del Valle L., Whitby D., Parsons C., Reiss K., Zabaleta J. (2015). Targeting HGF/c-MET induces cell cycle arrest, DNA damage, and apoptosis for primary effusion lymphoma. Blood.

[37] Derksen P.W.B., de Gorter D.J.J., Meijer H.P., Bende R.J., van Dijk M., Lokhorst H.M., Bloem A.C., Spaargaren M., Pals S.T. (2003). The hepatocyte growth factor/Met pathway controls proliferation and apoptosis in multiple myeloma. Leukemia.

[38] Konturek P.C., Konturek S.J., Sulekova Z., Meixner H., Bielanski W., Starzynska T., Karczewska E., Marlicz K., Stachura J., Hahn E.G. (2001). Expression of hepatocyte growth factor, transforming growth factor alpha, apoptosis related proteins Bax and Bcl-2, and gastrin in human gastric cancer. Aliment. Pharm..

[39] Rahman A., Pallichankandy S., Thayyullathil F., Galadari S. (2019). Critical role of H2O2 in mediating sanguinarine-induced apoptosis in prostate cancer cells via facilitating ceramide generation, ERK1/2 phosphorylation, and Par-4 cleavage. Free Radic. Biol. Med..

[40] Kitamura S., Kondo S., Shinomura Y., Kanayama S., Miyazaki Y., Kiyohara T., Hiraoka S., Matsuzawa Y. (2000). Met/HGF receptor modulates bcl-w expression and inhibits apoptosis in human colorectal cancers. Br. J. Cancer.

[41] Lim H.M., Lee J., Nam M.J., Park S.-H. (2021). Acetylshikonin Induces Apoptosis in Human Colorectal Cancer HCT-15 and LoVo Cells via Nuclear Translocation of FOXO3 and ROS Level Elevation. Oxidative Med. Cell. Longev..

[42] Lim H.M., Park S.H., Nam M.J. (2021). Induction of apoptosis in indole-3-carbinol-treated lung cancer H1299 cells via ROS level elevation. Hum. Exp. Toxicol..

